# Ophthalmology

**Published:** 1898-03

**Authors:** Cyril H. Walker


### OPHTHALMOLOGY.

Two years ago when **the Röntgen rays** had but recently been discovered it was predicted that, amongst other uses, they would be a help **in determining the presence of foreign bodies in the eyeball.** Attempts at first were unsuccessful, but during the past year considerable success has been achieved. De Schweinitz[3] related a case before the Ophthalmic Section of the College of Physicians, Philadelphia, in which he had twice attempted the extraction of a piece of steel with the electro-magnet and failed: on the twelfth day after the injury the fragment was located by the X-rays, extracted, and good vision resulted. Two cases are recorded[4] of the presence of a foreign body in the lens being determined by this means, and removed with good results some months after the injury. A

[3] *Am. J. M. Sc.*, 1897, cxiii. 566; abstract in *Ophth. Rev.*, 1897, xvi. 94.

[4] *Ophth. Rev.*, 1897, xvi. 396.

similar case is one in which a chip of steel had been in the vitreous chamber for no less than eighteen months. It is well-known how easily foreign bodies become "lost" in the orbit. Here again we find records of the successful use of skiagrams; a lead pellet, a fragment of a percussion cap, and a piece of steel embedded eight months are examples of this. Kibbe[1] relates three cases in which a foreign body was diagnosed, located, and removed; and a fourth case in which the fragment had glanced off the cornea and lodged in the eyelid. Owing to the œdema of the lid, it might otherwise have escaped detection for an indefinite period. Considering that it is frequently impossible to diagnose the presence of a foreign body in the globe by means of the ophthalmoscope, and the immense importance of an accurate diagnosis, all the skiagrams that can be obtained will be of value. It would undoubtedly be of service if all those in whom there was a probability of there being a fragment in the globe, even though it might have been quiet for months or years, could be subjected to the Röntgen rays under the existing improved conditions: radiography would make further advances, and with the increased statistics, the risk of a foreign body remaining in the eyeball could be more correctly estimated. It would not of course follow that if a fragment were detected any operation would be advisable. There can be little doubt that there are many cases in which a chip of steel has entered the eye, and a correct diagnosis was originally made, but eventually abandoned owing to the unexpected subsidence of symptoms.

In the **technique** of the process various methods have been adopted. Some have simply bandaged a rapid plate on the corresponding temple and then projected the rays transversely, but directed slightly backwards; others have used a narrow plate pushed as far as possible into the inner canthus and aimed the rays from the corresponding temple. But by far the best method is that of Mackenzie Davidson[2] by which not only the position but the approximate size of the foreign body can be ascertained.

I have seen reports of some twenty-five cases in which the Röntgen rays were used with success. Though in some instances there was loss of hair on the exposed temple, there was in **no case any serious result.** Chalupecký[3] has made some interesting experiments on this subject. Up to a certain point there is an analogy between the ultra-violet rays and those of Röntgen, and he thought it likely that the latter would have some effect upon the sensitive structures of the eye. It has been found that though the cathode rays are invisible in the ordinary sense, they still produce a sensation of light. This is apparently due, not to interruption of the rays by the lens and therefore fluorescence of that structure, but to the retina being

[1] *Arch. Ophth.*, 1897, xxvi. 517. [2] *Brit. M. J.*, 1898, i. 10, 372.
[3] See *Ophth. Rev.*, 1897, xvi. 336.

rendered fluorescent and so stimulating itself. Chalupecký's experiments on rabbits in May last show that prolonged exposure —some twenty-four hours—to the X-rays produced congestion and inflammation of the superficial structures; succeeded in a few days by baldness of the side of the head towards the Crookes's tube, and acute inflammation of the cornea followed by dense grey opacity. The effect, in short, was similar to that of some active chemical irritant. The other side of the head was in no way affected and the general health of the animal remained good. Casey Wood refers[1] to an instance in which a patient's head was submitted to the rays for about an hour, with the result that he lost a portion of his scalp and most of the surrounding hair. I cannot, however, find any single instance recorded of any serious injury being done in the attempt to radiograph a foreign body in the eyeball.

The literature of **Glaucoma** still grows apace. Czermak suggests that the increase of tension may in some cases be due to a shallow anterior chamber which is consequent on diminished secretion of the aqueous. In favour of this view he adduces the fact that the ciliary processes (which secrete the aqueous), are atrophied in the senile eye. Priestley Smith, reviewing Czermak's paper[2] points out the fact that the anterior chamber is very slowly re-established after iridectomy for glaucoma; that blood in the anterior chamber is very slowly absorbed under such conditions; and that the deterioration of vision after operation without further increase of tension points to degeneration of the secreting organs. A good many cases have been recorded in which taxis has proved beneficial. Several observers have seen serious harm from the use of cocaine in glaucoma; but Groenouw (Breslau) advocates its use, not as a substitute for iridectomy, but as a remedy in those cases in which eserine fails. He related three cases of acute glaucoma and two of absolute glaucoma, in which cocaine was successful. I have never ventured to rely on it in acute or subacute glaucoma, but a weak solution of it in conjunction with weak eserine often acts better than eserine alone. In absolute glaucoma it is of course perfectly safe to use cocaine, and I have frequently found it beneficial. Its good effect is probably partly due to the temporary contraction of the vessels of the iris and ciliary region which it produces. Weill, of Zürich,[3] attempted to set up glaucoma in the eyes of rabbits; but, like the experiments of Mauthner, his results were negative, yet his theory remained unshaken. Assuming, therefore, that glaucoma is due to irritation of the sympathetic, he advocates excision of a portion of the cervical sympathetic.

[1] *Ophth. Rec.*, 1898, vii. 44. [2] *Ophth. Rev.*, 1897, xvi. 201.
[3] *Am. J. Ophth.*, 1898, xv. 12.

The most recent statistics show that early iridectomy still gives the best results.

**Attempts to render the conjunctival sac aseptic** have not been successful. Randolph (Baltimore)[1] found germs in the conjunctival sac in 80, out of 100 healthy subjects. Of these 80, half were treated with aseptic irrigation and half with 1 to 5000 corrosive sublimate solution. The irrigation was thorough and was repeated three times in each case. Cultures were then made, and it was found that the aseptic and antiseptic treatment were equally futile. Only about 20 per cent. of the conjunctival sacs were rendered sterile. In almost every case the micro-organism found was one closely related to the staphylococcus pyogenes albus, but with much less pathogenic activity, and identical with the staphylococcus epidermidis described by Welch as a regular inhabitant of the skin and hair follicles. Trousseau found that after irrigating the conjunctival sac with 1 to 2000 sublimate lotion or 1 to 1500 cyanide of mercury—the strongest germicides the cornea will stand—the conjunctiva remained contaminated with bacteria. This perhaps explains the disappointing results obtained from the use of the perchloride in ophthalmia neonatorum: it apparently causes much smarting and is not more efficacious than boric acid lotion.

The **sterilisation of ophthalmic instruments** without either blunting or tarnishing them has always been a difficult problem. De Schweinitz [2] makes a useful suggestion. He finds that ten minutes immersion in a 1 to 1000 formaldehyde solution is satisfactory; but he prefers the use of pure formaldehyde gas. The instruments are first washed and dried, and are then placed in a box in the bottom of which there is a small dish of formaldehyde. A small piece of calcium chloride is placed in the dish and the close-fitting cover adapted. In ten minutes the instruments are sterile: no harm is done by leaving them in the gas for hours.

Two new local anæsthetics are introduced to our notice. **Holocaine** is a derivative of para-phenetidin. It is a white crystalline powder, insoluble (or sparingly so) in water; more soluble in ether, alcohol, or hot water. It is not necessary to sterilise solutions of the hydrochloride, as holocaine is directly bactericidal in its action. It causes smarting when first applied, but unlike cocaine it produces redness of the conjunctiva which may last an hour. A 1 per cent. solution possesses the following advantages over cocaine:[3] its action

[1] *Arch. Ophth.*, 1897, xxvi. 379. [2] *Ophth. Rec.*, 1897, vi. 380.
[3] *Ibid.*, 515.

is very rapid, anæsthesia being produced in fifteen seconds; it causes no dimness of the corneal epithelium; the effect is more lasting, anæsthesia being kept up for an hour after a few applications; it does not dilate the pupil, nor cause dimness of the cornea; its effect extends deeper than that of cocaine and causes anæsthesia of the iris even in glaucoma; it is absorbed by, and acts on, inflamed surfaces; it is always not only aseptic but antiseptic; it produces no toxic effects. It is relatively cheaper than cocaine and keeps better. It has been used in almost every variety of ophthalmic operation, and its only disadvantage is that it produces injection of the cornea and consequent bleeding in some operations. It produces a toxic effect if injected hypodermically.

**Orthoform**[1] is a white powder with no smell, slightly soluble in water and therefore slowly absorbed. It seems likely to be of great use in general surgery, and from its description should be of use in some varieties of chronic painful inflammations of the cornea. Applied to the eye of the rabbit it causes some redness and then anæsthesia.[2] It is said to be an antiseptic and in the form of fine powder or ointment it might prove of use in trachoma, but we can find no record of its introduction into ophthalmic surgery as yet.

Numerous diseases of the eye are the result of **auto-infection**, and Panas (Paris) has dealt with this subject recently, and points out many possible sources of infection which may easily be overlooked and on which ordinary text-books are silent. We quote the following from a review of the work by Priestley Smith:[3] "A common source of auto-infection of the eye through the medium of the blood is gonorrhœa of the genitals. The conjunctivitis which arises in this way is to be distinguished from that caused by direct contact with gonorrhœal pus. The discharge is rather mucous than purulent, the chemosis less severe, complications on the part of the cornea are less frequent and the whole course milder. The eye infection alternates with invasion of the joints or tendons. The same poison is a well-known cause of iritis, peripheral neuritis, sometimes of the optic nerve, tenonitis of the eyeball, and adenitis of the lacrymal gland."

Mild cases of the affection of the conjunctiva similar to that described above occur in Bristol, and I have met with patches of tenonitis in conjunction with, and presumably dependent on, gonorrhœa on several occasions.

CYRIL H. WALKER.

[1] *Brit. M. J.*, 1898, i. 362. [2] *Med. Chron.*, 1897-8, N.S. viii 199.
[3] *Ophth. Rev.*, 1897, xvi. 176.

